# Face Dependence of Schottky Barriers Heights of Silicides and Germanides on Si and Ge

**DOI:** 10.1038/s41598-017-16803-6

**Published:** 2017-11-30

**Authors:** Hongfei Li, Yuzheng Guo, John Robertson

**Affiliations:** 1 Engineering Dept, Cambridge University, Cambridge, CB2 1PZ UK; 2 College of Engineering, Swansea University, Swansea, UK

## Abstract

Density functional supercell calculations of the Schottky barrier heights (SBH) of metal germanides and silicides on Si or Ge find that these vary with the facet, unlike those of elemental metals. In addition, silicides and germanides show a stronger dependence of their SBHs on the work function than those of elemental metals, as seen experimentally. Both effects are beyond the standard metal induced gap states model. NiSi_2_ is found to have a much lower SBH on n-Si(100) than on n-Si(111), as seen experimentally. It is shown how such results can be used to design lower SBH contacts for n-Ge, which are needed technologically. The SBHs of the better behaved Si/silicide interfaces can be used to benchmark the behavior of the less well behaved Ge-germanide interfaces for this purpose. The dependence of the SBH of epitaxial Pb-Si(111) on its reconstruction is also covered.

## Introduction

For both conventional semiconductors and layered semiconductors, the performance of their devices is often limited by their contact resistances^1^. This is due to the Schottky barriers at the contacts. A standard way to minimize the Schottky barrier heights (SBH) is to vary the contact metal, as the SBH will vary with the metal work function. However, this is difficult for many semiconductors as they suffer from Fermi level pinning, in which the SBH varies only weakly with the work function. This is expressed in terms of the Fermi level pinning factor S = ∂ϕ_n_/∂Φ_M_ being very small, where ϕ_n_ is the n-type barrier height and Φ_M_ is the work function of the contact metal. For Si, the Fermi level pinning problem and a large SBH can be circumvented by heavily doping the Si, so that carriers can tunnel through the depletion layer, but this is less easy for other semiconductors where dopants are less soluble or not always shallow.

For example, germanium has higher electron and hole mobilities than Si and it is one of the more promising candidates for a next generation channel material. However, for Ge the n-type SBH is very large because the Fermi level is pinned close to its valence band edge^2,3^. Many approaches have been tried to overcome this problem, such as introducing ultra-thin oxide layers^4^, or delta-doping with As, S or Cl^5^. Another method is to use germanides or silicides^6–12^ which appear to have weaker Fermi level pinning^13^.

Schottky barrier behaviors are often classified as being limited by intrinsic properties^14–17^ or by extrinsic effects such as interfacial defects^18^. Generally, in Si or Ge the intrinsic effects tend to dominate. The intrinsic states include the metal induced gap states (MIGS)^14^. The MIGS are extensions of the travelling wave states of the metal continued into the band gap of the semiconductor and are drawn from the valence and conduction states of the bulk semiconductor^14,17^. As such, in a cubic semiconductor, the pinning factor S should be a function only of the semiconductor itself and *independent* of the type of metal, while the pinning energy or charge neutrality level (CNL) should be *independent* of the crystal face. Generally, if extrinsic effects are present, such as the interfacial defects at the contacts on MoS_2_, these tend to reduce S below the intrinsic MIGS value^19–21^ and so they are viewed as a negative factor.

However, there is a second type of ‘extrinsic factor’ in which varying the *type* of metal and the face can also be used to vary the Schottky barrier height. If this extrinsic quality can be accessed in a suitable way, it might be used to reduce the SBH of n-Ge.

It was previously noted that the SBHs of silicides on Si were much more weakly pinned than those of elemental metals^13^ – that is, S is much larger. Recently Nishimura *et al*.^22^ found that the SBH of germanides on Ge were also more weakly pinned than for elemental metals. They also found that their SBHs on Ge were face-dependent, whereas the SBHs of elemental metals were independent of face^3^. This is reminiscent of the behavior of NiSi_2_ where some years ago Tung^23^ found that the Si/NiSi_2_(111) interface had very different SBHs for its A and B orientations. Thus, here we carry out a detailed study of the face dependence of Schottky barrier heights of different metals.

## Results

**Figure 1:**
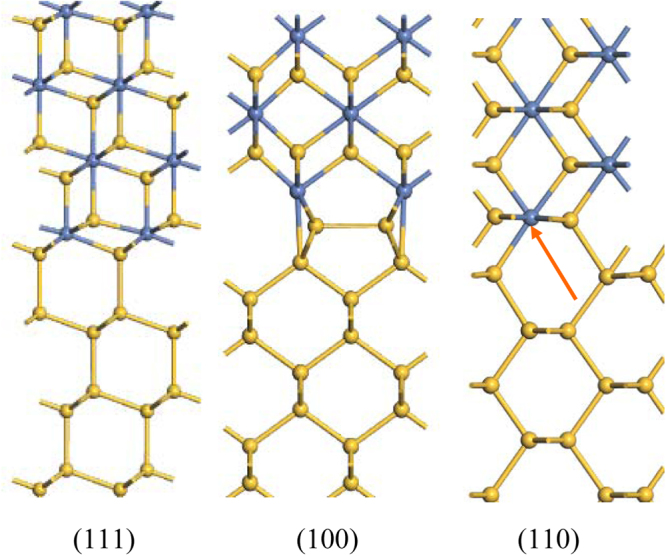
The atomic structure of (111), (100), and (110) Si/NiSi_2_ interfaces.

We first consider the disilicides. For silicide metals of higher work function, we use the cubic NiSi_2_ structure as a representative structure, as this has a high symmetry and is lattice-matched to Si. The Si/NiSi_2_ interface is interesting because the covalent bonding of the Si continues across the interface into the silicide. In NiSi_2_, the Ni sites are 8-fold coordinated by Si, and Si sites are 4-fold coordinated by Ni^23,24^. The bonds in NiSi_2_ themselves are only weakly polar^13^. At the interface, the NiSi_2_ lattice terminates in 7-fold Ni sites, which can be viewed as Ni ‘dangling bonds’, while the Si sites all remain 4-fold bonded across the interface^24^, Fig. 1(a). The Ni can be replaced by other transition metals to form their silicides. This NiSi_2_ structure is used for Ti, Cr, Fe, Ru, Co, Ni and Pt.

The (100) interface of NiSi_2_ has been much less studied than (111). Its structure was first modeled as containing 6-fold coordinated Ni sites^25^, but it was then noticed that it possessed a 2 × 1 reconstruction^26^. On the basis of total energy calculations, Yu *et al*.^27^ proposed an unusual 2 × 1 structure (Fig. 1(b)) which was later confirmed by Falke *et al*.^28^ using high resolution STEM. The 2 × 1 reconstructed interface has 5-fold Si sites and lateral Si-Si bonds on the Si side.

**Figure 2:**
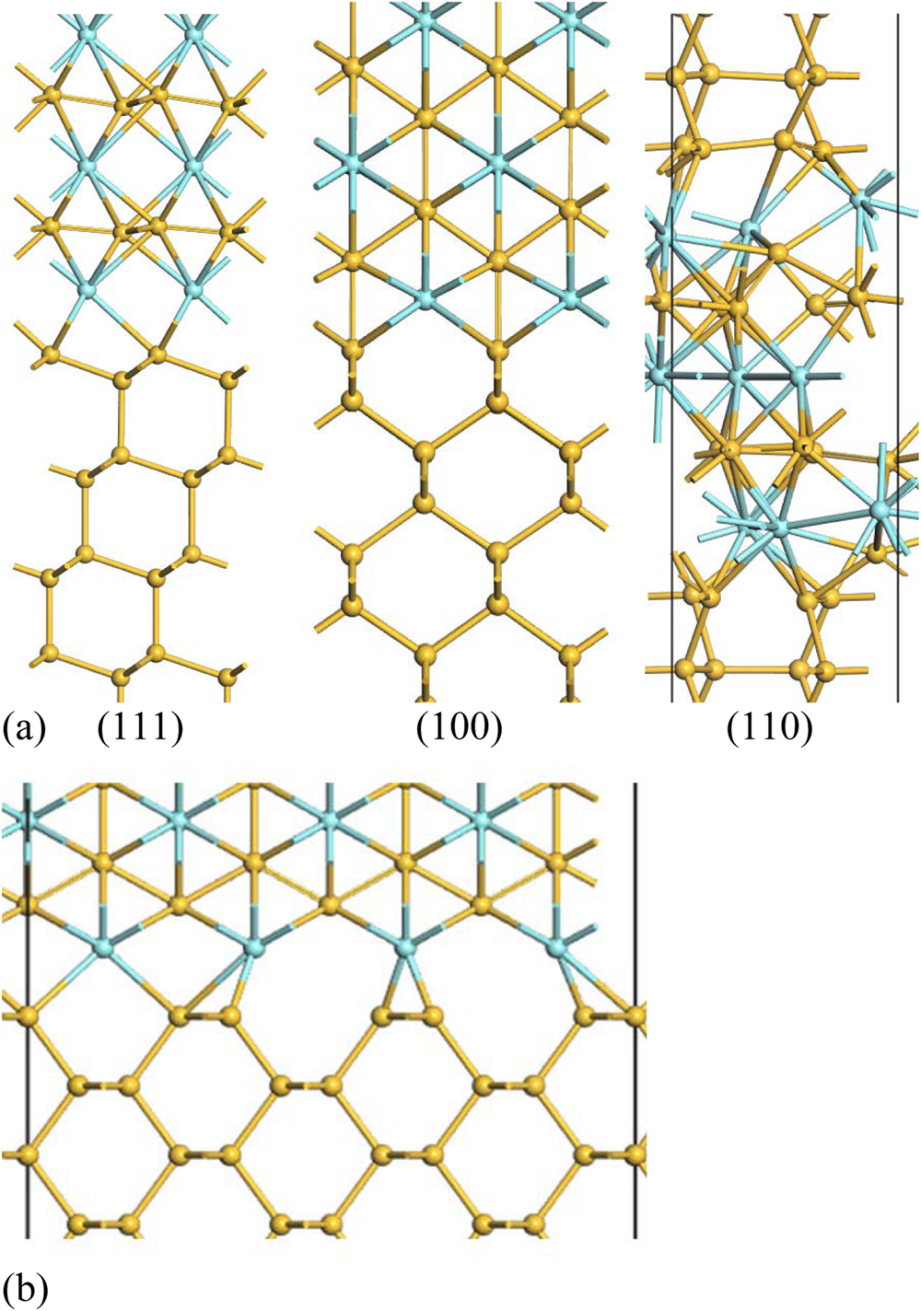
(**a**) Atomic structure of (111), (100), and (110) Si/YSi_2_ interfaces; the (110) Si/YSi_2_ interface with a disordered YSi_2_ layer. (**b**) The large (110) Si/YSi_2_ interface, not used in the calculations.

For silicides of metals of larger atomic radius, we use the YSi_2_ structure. This has a hexagonal unit cell which is lattice-matched to the √3 × √3(111) face of Si^11^, Fig. 2(a). Here, the silicons lie in a plane and the Y’s form layers above and below the silicons. The bonding in this lattice is very polar. The Si-terminated (0001) face of YSi_2_ bonds to Si(111). While YSi_2_ is hexagonal, its *a* and *c* lattice constants are quite similar. This allows us to construct a pseudo-(100)Si/YSi_2_ interface by rotating the YSi_2_ lattice to give the structure shown in Fig. 2(b). This structure is used for the silicides of Yb, Y, La and Zr. Thus, using NiSi_2_ and YSi_2_ lattices, we have the ability to study silicide/germanide interfaces from Yb to Pt, covering the widest range of metal work functions.

The structure of the (110)Si:NiSi_2_ interface is unknown, but a simple coherent interface model shown in Fig. 1(c) is used to represent this interface. We also built a (110) interface of YSi_2_ using the lattice matched interface shown in Fig. 2(b). However, this model has too large a lateral size and leaves too many dangling bonds on the Si side, and thus has a large interfacial energy. Instead, we took a slab of (110)YSi_2_ and disordered it by a molecular dynamics quench. This was then attached to the (110) face of Si and structurally relaxed. The resulting interface is shown in Fig. 2(c). It has no Si dangling bonds. This allowed us to study (110)Si/MSi_2_ interfaces for the full range of silicides using a combination of the NiSi_2_ and YSi_2_ structures. The resulting SBHs for the two types showed a continuous line of SBH values and reasonably well-behaved barrier heights.

**Figure 3:**
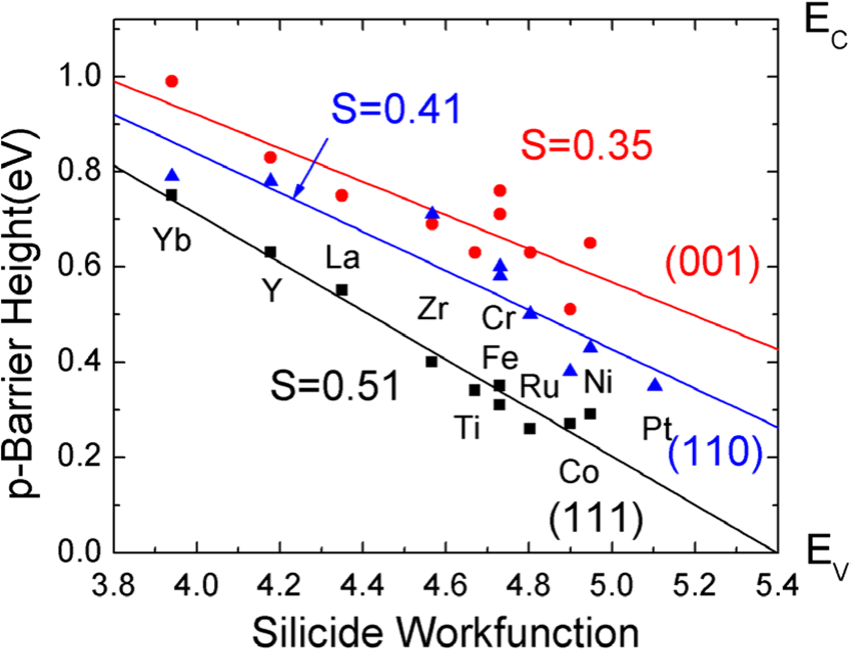
Calculated p-type Schottky barrier heights for Si/metal silicides, for (111), (110) and (100) interfaces, by the density functional supercell method.

The φ_n_ values for each face are plotted against the silicide work function in Fig. 3. This work function is estimated from the work function of the parent metal^29^ using a Miedema average^6^, WF = (Φ_M_Φ_Si_^2^)^1/3^. We see that the calculated φ_n_ values in Fig. 3 lie on separate lines for the (100) and (111) faces. We note the continuity of data points for NiSi_2_ and YSi_2_ lattices indicates that the φ_n_ values depend on work function and not the precise crystal structure. The (100) values are offset upwards from the (111) points. The calculated slope is S = 0.35 for (100) faces, and S = 0.51 for (111) faces. The slope for the most complicated interface (110) is found to be 0.41.

On average, the slopes are large, of order S~0.4, compared to those of elemental metals which are very small (S~ 0.05). This shows that silicide SBHs have much weaker Fermi level pinning than the SBHs of elemental metals. The calculated slopes for silicides are consistent with the experimental data^6,10^.

**Figure 4:**
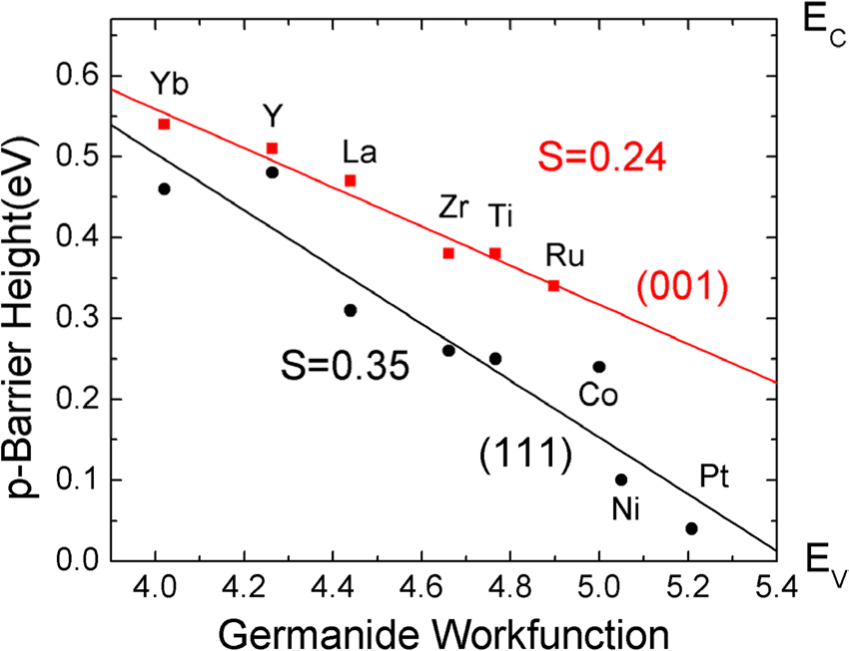
Calculated p-type Schottky barrier heights for Ge/metal germanides, for (111), and (100) interfaces, by the density functional supercell method.

A similar set of calculations were carried out for germanides on Ge. Due to the narrower band gap of Ge, and the band gap under-estimation of local density formalism, it is more difficult to locate the band edges of Ge, and there is greater uncertainty in the SBH values for Ge systems. Nevertheless, we see that there is a similar behavior for the Ge systems. A slope of 0.24 is found for the (100) face of germanides and a slope of 0.35 for the (111) faces, Fig. 4. This means that the barrier heights of germanides also show weaker Fermi level pinning, like the silicides. This gives rise to smaller SBH values for the electropositive germanides on n-type Ge, as in experiment^17^.

It is this large slope factor which allows the dependence of SBH on face to occur for silicides and germanides. They are both an ‘extrinsic effect’. Our calculations^13^ accounted for the well-known change in SBH from the Si/NiSi_2_(111)A to (111)B interface orientation seen by Tung^23^. They now explain the less well- known but *much larger* (0.4 eV) reduction in n-type SBH from Si/NiSi_2_ (111)A to (100) also seen by Tung^30,31^.

**Figure 5:**
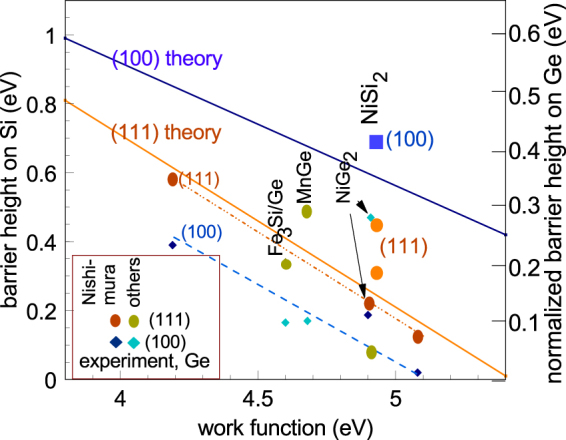
Normalised calculated barrier height values on Si and Ge from Figs 3 and 4, compared to experimental data for barrier heights from refs^12,22,30,32–36^.

Our calculated barrier heights for (100) facets lie above those for (111) for *both* Si and Ge. This is very clear if we normalise the Ge band gap and φ_n_ values to those of Si, as is done in Fig. 5. Our calculated results agree with experiment for Si/silicides, whose interfaces are abrupt, well-controlled and well characterised. However they are mostly opposite to experiment for Ge, where (111) SBH values often lie above (100) values as seen the experimental points in Fig. 5 for Nishimura^22^, Yamane^32^, Nishimura^33^ and Deng^34^. This suggests that Ge/germanide interfaces might be a problem, being disordered, non-abrupt or multi-faceted.

Experimentally it is more difficult to grow epitaxial germanides on Ge^32–36^. This is partly because of missing phases in the phase diagrams of bulk germanides^35^ and partly because of poor texture control and micro-crystallinity, which makes their Schottky barriers inhomogeneous and shifts the barrier size. Thus there are fewer well behaved epitaxial Ge/germanide systems for growth^33,34^.

Comparing experimental data with our calculated values in Fig. 5, we see that experimentally the (111) SBHs lie above the (100) values for the Ge/MnGe_x_ system^33^, for the range of germanides studied by Nishmura and Toriumi^22^ and for Fe_3_Si on Ge^32^. In some cases, one facet has an epitaxial layer on it while the other has a polycrystalline layer. On the other hand, there is the useful case of laser-processed NiGe_2_ where φ_n_ for the (100) facet is only 0.37 eV, compared to 0.6 eV for NiGe itself^36^. This case is consistent with our calculations. This is a reasonable starting point to create low ϕ_n_ SBHs for Ge. Thus, theory can help resolve some problems in these new channel materials.

**Figure 6:**
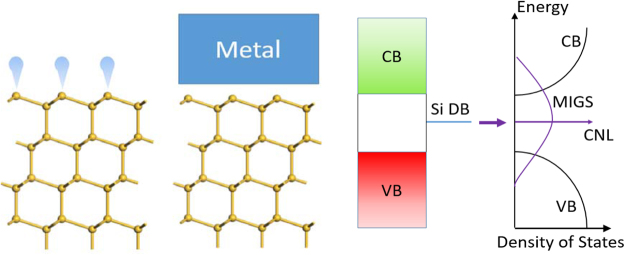
(**a**) Schematic of (111) Si surface, showing Si dangling bonds, (**b**) Si/metal interface, (**c**) schematic band diagram with the Si dangling bond states having broadened into MIGS.

What is the cause of the different behavior of elemental metals and silicides? At the Si surface, there are Si dangling bonds. When this Si surface makes an interface with an elemental metal, these dangling bond (DB) states hybridize with the metal states and form the MIGS. These MIGS spread out in energy across the whole bonding-antibonding gap of the Si^15^. The average energy of the MIGS or the CNL energy is therefore that of the Si DB states from which they originated, Fig. 6. Thus, all the properties of these MIGS are intrinsic to the semiconductor.

**Figure 7:**
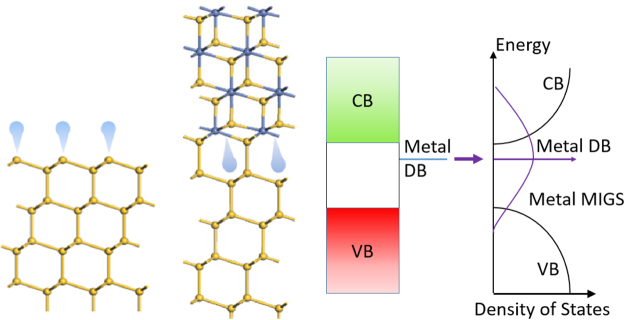
(**a**) Comparison of Ni dangling bonds at a silicide interface, (**b**) how these Ni DB states broaden out to form MIGS at a silicide interface, and (**c**) whose average energy varies as you change the metal.

Now, at a Si/silicide interface, and taking the Si/NiSi_2_ interface as an example, the Si-Si bonds continue across the interface. There are no Si DBs on the Si side pointing into the metal region, the dangling bonds are on Ni atoms on the silicide side. Indeed, the state at E_F_ for NiSi_2_(111) interface causing the SBH shift was shown to have Ni DB character by Lin^13^. When the interface forms, these metal DB states spread out in energy across the Ni-Si bonding-antibonding energy gap to form the MIGS, Fig. 7. The average energy of these MIGS is now the average energy of the Ni-Si energy gap, or E_x_ = ½(E_M_ + E_Si_) in general, where these energies are the work functions of the metal and Si respectively. Hence when the silicide’s metal changes, the average energy of the MIGS also changes, by ½ the rate of E_M_. The slope factor S to zeroth order will therefore be ~0.5, as indeed it is.

**Figure 8:**
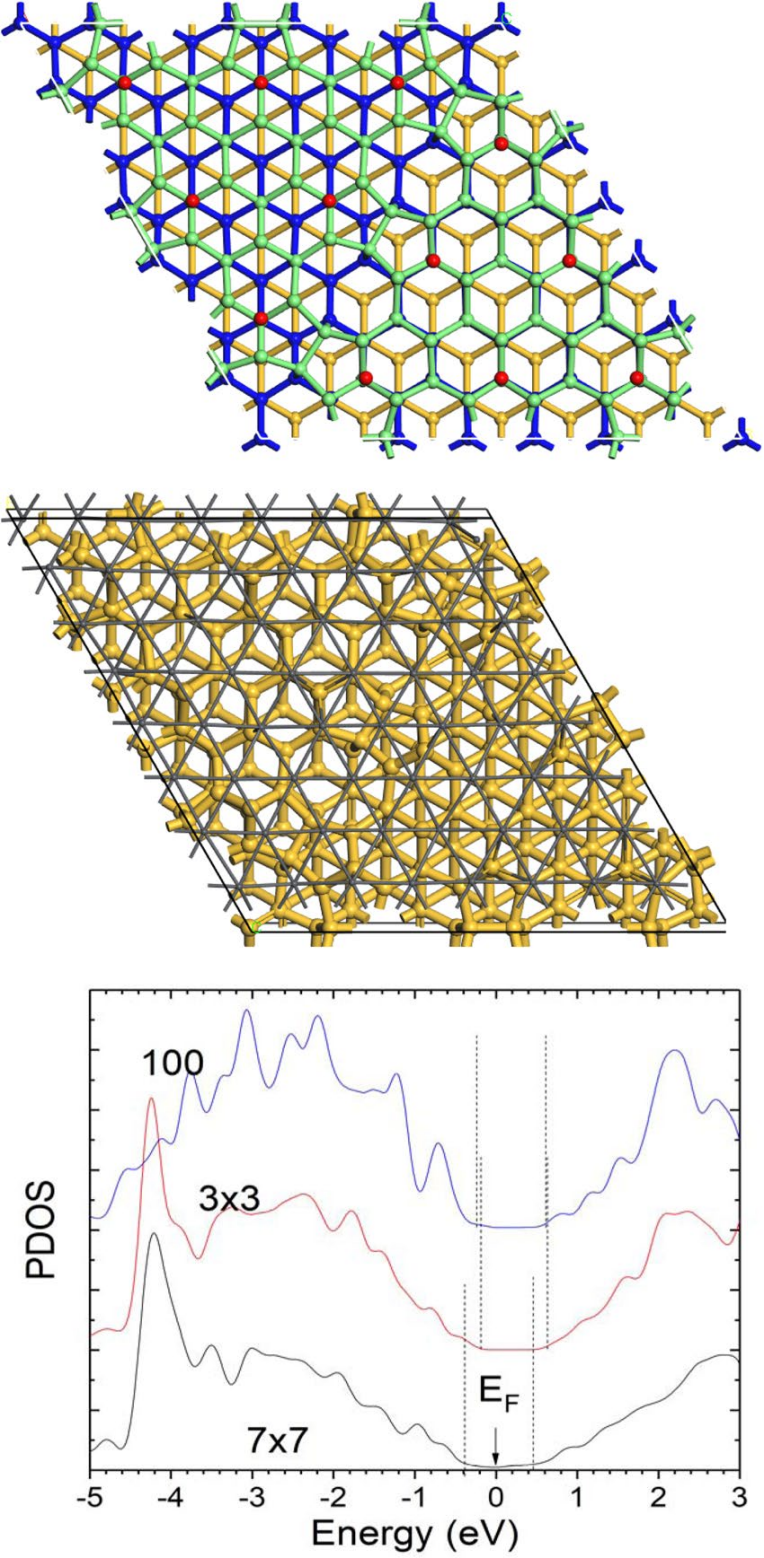
(**a**) Si(111)7 × 7 structure with different layer heights colored. (**b**) Positioning of Pb atoms over the Si sites for the first Pb layer. (Pb = small grey balls). (**c**) Calculated local density of states near the band gap, vertical lines showing the band edges, for the two (111) interfaces and the (100) interface with √3 × √3 Pb layer.

We finally consider the Pb/Si epitaxial system where a structure-dependent SBH was found experimentally^37,38^. In this case Pb is an elemental metal. In this case, room-temperature deposited Pb on the 7 × 7(111)Si face^39,40^ shows a smaller 0.7 eV SBH on n-Si. When this is annealed, the interface changes to a rotated √3 × √3 lattice matching. This has a larger 0.93 eV SBH on n-Si experimentally. We have calculated the SBHs from supercells of these structures. The SBHs extracted from the Si band edges of the partial density of states at bulk Si layers referenced to E_F_ in Fig. 8 show a similar shift to experiment, with a larger n-type SBH for the √3 × √3 phase. It has been remarked that the √3 × √3 system has an unusually large n-type SBH^37^, well below the trend of other elemental metals on Si. We see that the calculated SBHs for the √3 × √3(111) and √3 × √3(100) are similar.

## Summary

In summary, the barrier heights of a wide range of metal disilicides and metal digermanides have been calculated using supercell models of the interfaces, for the three higher symmetry faces, (100), (111) and (110). The barrier heights show that the SBHs have a dependence of crystal facet, and much weaker Fermi level pinning than for elemental metals, which goes beyond MIGS theory. The dependence on facet is consistent with data for the (111) and (100) interfaces of NiSi_2_ and can be used to help understand the more complicated behavior of the facet dependence of germanides on Ge. These effects can be used to tune the SBHs for n-type contacts on Ge, which is presently a significant impediment to the use of Ge high mobility substrates in future CMOS devices.

## Methods

We have calculated the SBHs of metal silicides and germanides on Si or Ge for metals of a wide range of work functions, using supercell models of the semiconductor and the silicide or germanide. The calculations are carried out using the plane wave density functional code CASTEP^41,42^. We use the generalized gradient approximation for the electron exchange-correlation functional, and norm-conserving pseudopotentials with a cutoff energy of 750 eV. The density of states calculations for Ge used a different pseudopotential which gives a band gap even in GGA, generated by OPIUM code. The convergence is carried out to an energy below 10^−5^ eV per atom, and with forces below 10^−3^ eV/Ǻ. A k-point mesh of 4 × 4 × 2 is used for Brillouin zone integrations. The calculations are carried out on supercells of 5 layers of silicide and 9 layers of Si (and no vacuum layer). The lattice geometry is relaxed in GGA. When there is a lattice mismatch between the Si and the silicide, the x, y lattice constants of the silicide are kept fixed to those of the Si and the vertical z distances are allowed to relax. This roughly conserves the silicide volume. The work function of a metal depends mostly on its atomic volume.

For the Si/Pb calculations, ultra-soft pseudopotentials were used with a 260 eV plane wave cutoff energy. There are 6 (double) layers of Si and 3(6) layers of Pb for Si 111 (100/110) interface. The Si (111) surface is built from the experimental structure reported in RefXX. A 5 × 5 supercell is used for (100) interface and a 3 × 3 supercell is used for (110) with a rotation of 45°. The lattice mismatch is less than 3% in all interface models. Only Γ point is used for reciprocal space integration in (111) model while 5 × 5 and 9 × 9 MP grid point is used for (100) and (110) respectively. The other side of Si slab is passivated with H. A 30 Ǻ vacuum is inserted in the model, which is confirmed by calculating the electrostatic potential to be thick enough to cut off the image interaction introduced by the periodical boundary condition.

The GGA functional is known to under-estimate the semiconductor band gap, thus it will also under-estimate the Schottky barrier height, as has been known for some years^43^. Here we can correct this effect by also calculating the density of states using the screened exchange (sX) hybrid density functional^42^. Generally though, we are interested in *changes* in SBH due to the face or *rates of change* of SBH with work function, which are not affected by the absolute band gap.
